# DNS Privacy Leaking Mitigation and Energy Costs: Measuring the Impact of Adding Dummy Queries on Encrypted DNS Protocols

**DOI:** 10.3390/s26206577

**Published:** 2026-10-16

**Authors:** Andrea Jimenez-Berenguel, Marta Moure-Garrido, Celeste Campo, Carlos Garcia-Rubio

**Affiliations:** Universidad Carlos III de Madrid, Departamento de Ingeniería Telemática, Av. de la Universidad 30, 28911 Leganés, Madrid, Spain; andrejim@pa.uc3m.es (A.J.-B.); celeste@it.uc3m.es (C.C.); cgr@it.uc3m.es (C.G.-R.)

**Keywords:** DNS privacy, encrypted DNS, energy measurement, traffic analysis, energy-efficient security

## Abstract

The Domain Name System (DNS) was originally designed without privacy mechanisms. Encrypted variants such as DNS over TLS (DoT), DNS over HTTPS (DoH), DNS over HTTP/3 (DoH/3), and DNS over QUIC (DoQ) protect the content of the queries, but the resolver and any network observer with access to the traffic metadata can still profile the user. Client-side defenses counter this by adding dummy queries to the real DNS traffic of the device. On a smartphone, the battery cost of such a defense has not been characterized. We evaluate the energy cost of adding dummy queries on an unmodified Android device using the BatteryStats subsystem. Since the dummy queries must be sent over the protocols the device already uses, we measure this cost for the five DNS protocols in use (Do53, DoT, DoH, DoH/3, and DoQ) and four public resolvers (Google DNS, Cloudflare, Quad9, and AdGuard DNS), at both the application and system-resolver levels. Our results show that the cost of the countermeasure depends on the protocol and resolver used, on the number of dummy queries added, and on their scheduling: sending them as a single burst rather than periodically reduces the energy cost for the same privacy gain.

## 1. Introduction

The Domain Name System (DNS) [1] was deployed over 35 years ago to provide, among other services, the translation of human-readable domain names into IP addresses, acting as the phonebook of the Internet. The original DNS implementation relies on the UDP transport protocol and transmits queries and responses in plaintext over port 53, without privacy or security protection. As an essential cornerstone of the Internet, DNS carries private information. However, the lack of protection leaves the original design vulnerable to a wide range of active and passive attacks, including surveillance, censorship, and traffic manipulation [2,3], which seriously compromise users’ privacy.

In response to these threats, different protocol extensions have been developed. In short, DNSSEC [4] digitally signs DNS records to ensure authenticity and integrity, preventing attacks such as DNS spoofing, but does not encrypt traffic. DNS over TLS (DoT) [5] encrypts queries and responses between the client and the resolver using TLS, preventing eavesdropping. DNS over HTTPS (DoH) [6] also encrypts DNS traffic but uses the HTTPS protocol over HTTP/2, making it harder to block or filter DNS requests. DoH/3 [7] encapsulates DNS queries within HTTP/3, which runs over QUIC, inheriting the anti-censorship properties of DoH while benefiting from QUIC’s reduced handshake latency and built-in encryption. DNS over QUIC (DoQ) [8] carries DNS queries directly over QUIC without an intermediate HTTP layer, combining the encryption of DoT with the speed of QUIC. Oblivious DNS over HTTPS (ODoH) [9] introduces a proxy between the client and the resolver so that no single entity knows both the client’s identity and the query content. However, encrypted DNS is not always enough to protect user privacy [10,11]. Encrypted DNS traffic remains vulnerable to traffic analysis attacks that can infer user activity from metadata such as packet sizes and timing [12,13]. Recent deep learning models go further and extract malicious signatures from high-entropy TLS 1.3 flows using only statistical and temporal flow features, without access to the encrypted payload [14]. Furthermore, there are few DNS servers that support encrypted DNS [15], which could lead to monopolization and centralization of DNS services where providers collect private information across services. Li et al. [16] found that 57.05% of DoH servers are operated by five organizations. Even with encrypted DNS, network observers and DNS servers can observe user habits to build user profiles [17]. The literature proposes privacy measures beyond encrypted DNS, including trusted computing environments [18], traffic obfuscation [19], collaborative query diffusion [20], private information retrieval [21], and the addition of dummy queries to real DNS traffic [17].

Among all the devices a person uses, the smartphone is the one that provides the most complete picture of their daily routine. Instant-messaging applications reveal when and with which services the user communicates, cloud-synchronized calendar applications reveal how their day is organized, and entertainment applications reveal leisure habits. The same device also runs applications belonging to sensitive categories, such as banking, health, or religion, whose mere presence in a traffic trace discloses vulnerable information about the user. Every one of these applications resolves domain names before it can exchange any data, so the DNS traffic emitted by a phone is an almost complete index of the activity taking place on it. This is the setting in which query-pattern obfuscation defenses are most valuable, and it is also the device on which such a defense has to be deployed. For this reason, we perform our measurements on a commercial smartphone rather than on a laboratory testbed.

Deploying such defenses on a smartphone is not free. Adding dummy queries consumes battery on a device whose energy budget is strictly limited, and where users are sensitive to any application that shortens the time between charges. The cost is not only individual: DNS resolution is one of the most frequently repeated network operations on the Internet, so a per-query overhead multiplied by billions of devices has a measurable aggregate footprint, which matters for the current push towards energy-efficient and environmentally sustainable networking. These protocol extensions are already known to increase latency and resource consumption across DNS variants [22,23], and the same effect has been observed for HTTP [24,25]. Quantifying how much battery encrypted DNS and the privacy mechanisms built on top of it actually drain on a phone is therefore a practical prerequisite for deploying them.

The primary goal of this paper is to measure the energy cost, on a mobile device, of a client-side countermeasure against DNS-based user profiling: adding dummy DNS queries to the real DNS traffic generated by the device, so that the real queries are camouflaged among the dummy ones and a network observer, such as a curious or compromised resolver, cannot tell them apart. In our previous work [17], we proposed one such Privacy Enhancing Technique (PET), DNS Query Forgery (DQF), which adds dummy DNS queries from the client side to hinder user profiling based on DNS traffic, at the cost of additional network overhead. DQF does not rely on third parties or server-side implementations to provide privacy. We stress that, throughout this paper, adding dummy queries is not an attack on encrypted DNS, but a defense applied by the user’s own device over its own DNS channel. Since the dummy queries can be sent over any of the DNS protocol variants that a mobile device generates today, we measure the power consumption of sending DNS queries with the five main variants currently in use (DNS over UDP (port 53) (Do53), DoT, DoH, DoH/3, and DoQ) across four major public DNS resolvers: Google DNS [26], Cloudflare [27], Quad9 [28], and AdGuard DNS [29]. The purpose of this comparison is not to rank the protocols per se, but to quantify how much energy the countermeasure costs depending on the protocol, the resolver, the schedule of the dummy queries, and the cache configuration with which it is deployed. The measurements are performed on a Google Pixel 8 Pro [30] running stock Android 15, using the BatteryStats subsystem through the Android BatteryManager API [31] as the energy measurement source. We additionally collect on-device traffic captures with PCAPdroid v1.9.0 (https://github.com/emanuele-f/PCAPdroid, accessed on 1 March 2026) and decrypt them using the TLS session keys exported by our application, in order to inspect the categories of packets exchanged by each encrypted DNS variant.

Our contributions are as follows:

- We quantify, on the battery of a real, unmodified Android phone, the energy cost of adding dummy DNS queries to the traffic of the device as a privacy countermeasure, for each of the five DNS protocols over which the dummy queries can be sent (Do53, DoT, DoH, DoH/3, and DoQ) and for four major public resolvers (Google DNS, Cloudflare, Quad9, and AdGuard), reporting both average power and resolution time.
- We analyze how the energy cost of the countermeasure depends on the number of dummy queries added within an observation window and on how they are scheduled, either emitted as a single burst or spread periodically across the window.
- We evaluate the platform mechanisms that an application adding dummy queries cannot control. We measure the Android system resolver under Private DNS, the protocol allowlist that determines which transport each resolver is given, and the shared system DNS cache, which reverses the relation between query rate and power.
- We explain why the cost of the countermeasure differs across protocols. Decrypting the captured traffic with the exported TLS session keys attributes the energy gap to handshake round trips, connection reuse, and keep-alive traffic rather than to packet volume, which is why the QUIC-based variants are cheaper even though they exchange more packets.

The remainder of this paper is organized as follows. Section 2 provides the background and reviews the related work: the evolution of DNS privacy protocols, the prior measurements of DNS protocol cost, and the Android energy measurement literature. Section 3 describes our measurement methodology, including the adversary model and the dummy-query countermeasure whose cost we measure, the testbed, and the custom Android application. Section 4 describes the open-world measurement conditions, presents the experimental evaluation, discusses its results, and states the limitations of the study. Finally, Section 5 draws conclusions and outlines future work.

## 2. Background and Related Work

This section provides the background required to interpret our measurements and positions them with respect to prior work. Section 2.1 briefly reviews the evolution of DNS privacy protocols. Section 2.2 reviews prior measurements of the performance and energy cost of DNS protocol variants, and Section 2.3 reviews energy measurement approaches for Android applications. The adversary model and the dummy-query countermeasure whose energy cost motivates this study are described in Section 3.1.

### 2.1. Evolution of DNS Privacy Protocols

DNS was originally designed as an open, unencrypted protocol. The growing awareness of privacy threats, including ISP-level traffic interception [32] and DNS-based censorship, has driven successive efforts to add confidentiality to DNS communications. Table 1 summarizes the security and privacy extensions to DNS standardized through the IETF RFC process.

The first extension, DNSSEC [4], targeted integrity rather than confidentiality: it signs records digitally but leaves the traffic in plaintext. Confidentiality arrived with DoT [5], which encrypts the interaction with TLS 1.3 on a dedicated port (853), and was pursued in parallel by the UDP-based DNS-over-DTLS [34] and by the non-IETF DNSCurve [35] and DNSCrypt [36], both based on Curve25519 [37], all of which remained largely experimental. QNAME Minimisation [33] complements encryption by reducing the query information disclosed to authoritative servers. DoH [6] then moved DNS onto HTTP/2 over TCP+TLS on port 443, making it indistinguishable from regular web browsing and therefore harder for middleboxes to block, and ODoH [9] added a proxy so that the resolver knows the query but not the client, and the proxy the client but not the query.

The latest generation replaces TCP+TLS with QUIC, which merges the transport and cryptographic handshakes into a single round trip. DoQ [8] carries DNS directly over QUIC on port 853, with no HTTP layer, whereas DoH/3 encapsulates it in HTTP/3 [7] and so inherits the HTTP semantics and the port-443 blending of DoH. This distinction between the TCP/TLS-based and the QUIC-based transports is the one that matters most for our measurements, since it determines how many round trips a resolution costs and therefore how long the radio stays active.

A further line of work is now adding post-quantum cryptography to DNS, either through DNSSEC signatures [38,39] or in the TLS layer underneath DoT [40], at a reported cost of 33–40% throughput loss. This confirms the trend that motivates our study: every new layer of DNS protection is paid for in computation and traffic, and therefore in energy.

In this paper we focus on the five variants currently used in practice for client-resolver communication: Do53, DoT, DoH, DoH/3, and DoQ. Their main differences in terms of transport, encryption layer, and default port are summarized in Figure 1, which shows the structure of the packet layers starting from the IP layer for each protocol variant.

### 2.2. Energy and Performance Costs of DNS Protocol Variants

The progressive addition of security layers to DNS introduces measurable overhead in terms of latency, CPU utilization, memory consumption, and energy. Table 2 summarizes the main studies that have quantified these costs.

We group the prior studies by the type of metrics they report. A first group measures *resource-level* metrics (CPU power, memory, and energy) at the resolver or server side. Renout et al. [23] found that DoH consumes about six times more CPU power than UDP-based DNS and DoT about three times more, which their model attributes to DoH requiring 25 packets per query versus two for cached UDP. Le Louët et al. [41] reported that moving a resolver from UDP to DoH costs 70% of its capacity in queries per second, and that under single-query connections DoH reaches 9.85 kWh/day against 0.05 kWh/day for UDP, a nearly 200-fold increase.

A second group measures *client-perceived timing*: resolution time, round-trip time (RTT), and page load time (PLT). Aydeger et al. [42] reported a 607% latency increase from DNS to DoH at an IoT edge deployment, and Chhabra et al. [10], measuring 22,052 residential clients in 224 countries, reported a median initial DoH query of 415 ms against 234 ms for Do53, although 28% of clients were faster with DoH. The picture is not uniformly unfavorable to encryption: Hounsel et al. [22] found DoT and DoH to yield faster page loads under loss, since TCP retransmission beats UDP timeouts, and Sengupta et al. [43] showed that the 30–50% PLT inflation of DoH is largely recovered by coalescing DoQ with HTTP/3 0-RTT on a single QUIC connection. Bielefeld et al. [44], in the first large-scale comparison of DoQ and DoH/3, found the PLT penalty of both to be within ±1% of Do53 across the top one million websites.

In contrast to these works, we expanded the scope of protocol evaluation and measured all five main DNS protocol versions (Do53, DoT, DoH, DoH/3, and DoQ) on a real Android device under realistic open-world conditions, combining device-side power measurements, per-query response time, and decrypted traffic captures analysis.

### 2.3. Android Application Energy Measurement

Measuring the energy consumption of mobile applications is a well-studied problem in the software engineering community. Approaches can be broadly categorized into hardware-based and software-based methods. Table 3 provides a comparison of the main tools and studies in the literature.

*Hardware-based approaches* rely on external meters. Zhang et al. [45] used a Monsoon FTA22D to build PowerTutor, a component-level power model accurate to within 6.27%; Aragón-Jurado et al. [46] used a Monsoon monitor to show that compiler optimizations for speed do not always reduce energy; and Wang et al. [47] built accurate power models for embedded devices from a consumer-grade UM25C USB meter.

*Software-based approaches.* The terms Batterystats, BatteryManager API, and BatteryStats API all refer, in practice, to the same Android subsystem: a kernel-level battery accounting service (batterystats) accessible from the application layer through the BatteryManager API and from adb via dumpsys batterystats/Battery Historian. Differences across studies lie mainly in which front-end is used to collect the data. The works that rely on this subsystem essentially read the same low-level quantities exposed by the device, namely the battery voltage, the instantaneous and average current draw, the remaining battery charge counter, and (when available) a cumulative energy counter. From these direct measurements, derived quantities such as instantaneous power (P=V·I), energy over an interval, or per-method energy attribution are computed offline by each tool.

The tools built on it differ mainly in granularity and purpose. PETrA [49,50] combines Batterystats with Systrace to attribute energy at method level, and was later used to locate energy hotspots in real applications [52]; PowDroid [51] offers the same accounting as a black-box tool requiring no source access. Others apply it comparatively: Horn et al. [53] measured native Android apps to consume on average 53% less energy than their web counterparts, Groza et al. [54] and Fawad et al. [55] used battery counters to detect and refactor energy code smells, achieving 4–30% drain reduction, and Guégain et al. [48] attributed battery discharge to individual hardware units on a Pixel 8.

In our work, we use the BatteryStats subsystem through the Android BatteryManager API [31] on a Pixel 8 Pro. The application directly reads voltage and current at a fixed sampling interval and computes instantaneous power as P=V·I, then integrates over the benchmark window to obtain the energy consumed during DNS activity. We characterize the power consumption of various DNS protocols and dummy-query scheduling strategies using different DNS servers. Unlike external meters, BatteryStats does not provide per-query energy, but it allows comparative measurements of the whole device under its real operating conditions, which is the quantity of interest when a privacy countermeasure is deployed on a phone.

## 3. Measurement Methodology

In this work we measure the energy cost incurred on a mobile device by a client-side countermeasure against DNS-based user profiling, which consists of adding dummy DNS queries to the real DNS traffic generated by the device in order to conceal the user’s query pattern. Since this cost is the cost of sending additional DNS queries, we measure it for the different versions of the DNS protocol (Do53, DoT, DoH, DoH/3, and DoQ) across four major public DNS resolvers. We first state the adversary model and the countermeasure whose cost we measure, and then describe the testbed, the custom Android application developed for query generation and energy logging, and the metrics collected.

### 3.1. Adversary Model and Dummy-Query Countermeasure

**Adversary model.** We consider a passive network observer whose goal is to profile the user from the DNS traffic generated by the device. Encryption hides the content of the DNS queries from third parties, but it does not prevent this profiling. On the one hand, the recursive resolver, for instance a curious or compromised one, receives every queried name in plaintext because it has to resolve it, and therefore observes the complete sequence of domain names requested by the device, together with their timing. On the other hand, any network observer on the path between the device and the resolver can still access the metadata of the encrypted DNS traffic, such as packet sizes or inter-arrival times, which are sufficient to infer the websites or services accessed by the user, as demonstrated for DoH [12,13] and, more recently, for DoQ [56]. In both cases, the adversary can build a profile of the user’s activity and interests without modifying the DNS traffic.

**Countermeasure.** To mitigate this privacy leakage, we proposed in our previous work DQF [17], adding dummy queries to the real DNS traffic from the client side. The dummy queries are added to the traffic of the device and form a stream parallel to the genuine queries of the applications, so the real queries and the traffic generated by the applications are neither modified nor intentionally delayed, dropped, or retransmitted. As a side effect, the real queries may be slightly delayed, since more queries have to be handled and, in a real deployment generating a large number of dummy queries, they may be queued. What the countermeasure directly increases is the use of the resources of the device and, therefore, its energy consumption. The adversary observes the combination of both streams and cannot distinguish the real queries from the dummy ones, so it obtains a distorted view of the user’s interests. The privacy gain is quantified in [17] as the Kullback–Leibler divergence between the real query distribution of the user and the distribution observed by the adversary: the larger the proportion of dummy queries, the larger the privacy gain and the larger the overhead. This is the mitigation referred to in the title of this work, and the energy overhead it introduces on the device is what we measure in Section 4.

In the following paragraphs we describe the testbed used to perform the measurements, the custom Android application developed for query generation and energy logging, and the metrics collected.

### 3.2. Testbed Architecture

The experimental setup consists of a mobile phone that communicates with different DNS servers and a computer acting as the development host for subsequent traffic analysis, as shown in Figure 2. The mobile device under test is a Google Pixel 8 Pro [30], equipped with a Google Tensor G3 processor, 12 GB LPDDR5X RAM, and a 5050 mAh battery. The device runs the factory-default Android 15 configuration as shipped by Google, with no root access and no software or hardware modifications. This choice was deliberate: we aimed to reproduce conditions as close as possible to those of an unmodified consumer device, rather than those of an instrumented laboratory platform. Nevertheless, all experiments were performed with a single device model, operating system version, mobile network, and location, so the absolute values reported here should not be taken as representative of all users; we discuss this limitation in Section 4.5. To reach the selected DNS servers and make queries using different DNS protocol versions, the device is provisioned with a SIM card.

For the energy measurements, we implemented a custom Android application, described in the following subsection, and compared the results across different DNS servers. For the DNS servers, we target four major public resolvers: Google Public DNS (8.8.8.8) [26], Cloudflare (1.1.1.1) [27], Quad9 (9.9.9.9) [28], and AdGuard DNS (94.140.14.14) [29]. Using multiple resolvers allows us to compare the measured energy cost across different server deployments. Quad9 and AdGuard were included because they support DoQ, whereas Google and Cloudflare do not currently offer DoQ.

To avoid charging current contaminating the measurements, the device remains disconnected from any cable during each experiment; the USB-C connection to the development host is used only between sessions to transfer the resulting data files.

### 3.3. DNS Benchmark Application

To measure the energy consumption of the different DNS protocol versions on the mobile device, we developed a custom Android application written in Kotlin. The application sends a configurable number of DNS queries to a selected target within a defined time window, and records energy metrics throughout the execution.

We obtain the energy-related parameters using the Android BatteryStats subsystem through the BatteryManager API [31], and store all the metrics collected during a benchmark run as a CSV file for offline analysis.

#### 3.3.1. Metrics

In the literature, DNS performance and energy consumption have been characterized through metrics such as CPU power and energy [23,41], latency or resolution time [10,22,42], page load time [43,44], and traffic volume. However, prior work typically compares only a subset of the available DNS protocol variants, and most measurements are performed on servers, virtualized clients, or controlled testbeds. In this study, we measure all five main DNS protocol variants (Do53, DoT, DoH, DoH/3, and DoQ) on a real Android 15 device under open-world conditions. We collect the metrics typically reported in the literature: the power consumed by the device and the response time of each query. Additionally, we also analyze the captured traffic using the corresponding SSLKEYLOGFILE.

**Power:** We are interested in the average power consumed by the device while DNS queries are being sent within a given observation window, since dummy queries can be added either as a single burst or in a periodic pattern. Individual DNS queries consume very little energy, and their impact cannot be isolated by the on-device sensors. The aim is therefore to detect any noticeable increase in power consumption rather than to obtain fine-grained per-request measurements: we modulate the query rate within the observation window and analyze the resulting average power, so that all results are reported relative to the query frequency. The application samples the variables voltage_now and current_now exposed by the BatteryStats subsystem once per second, and computes the per-second instantaneous power as

(1) P(t)=|I(t)|×V(t),

where I(t) is the current in mA and V(t) is the battery voltage in V [57]. The reported power for a benchmark window is the average of the per-second samples.

**Resolution time:** For each query, the application records the resolution time as the elapsed time from sending the DNS query to receiving the response, measured using System.currentTimeMillis(). The resolved addresses are discarded immediately after recording the resolution time, ensuring that post-processing does not influence the timing. This method returns wall-clock time with a granularity of 1 ms and can be adjusted by network time synchronization during a run, unlike a monotonic source such as System.nanoTime(). Since the measured resolution times are in the order of tens to hundreds of milliseconds (Section 4.2.2 and Section 4.3.2), the 1 ms granularity is negligible, and a clock adjustment would produce an isolated outlier rather than a systematic bias between configurations. Future versions of the application will use a monotonic timing source.

**Decrypted traffic:** To inspect which categories of packets are actually exchanged by each encrypted DNS protocol, we collect on-device traffic captures analogously to the energy benchmarks rather than simultaneously, since running a capture during a power benchmark would itself consume additional energy and contaminate the readings. The captures are performed with PCAPdroid, filtering by application (our custom benchmark application), and stored as .pcap files. Using the SSLKEYLOGFILE exported by the application during the corresponding run, the captures are then decrypted and grouped by packet category (DNS request/response, TLS handshake, ACK, control, ping, QUIC stream, etc.) for visualization purposes.

#### 3.3.2. Application Modes

The application separates the measurements into two categories: *app-level measurements*, where the application directly implements each DNS protocol variant and selects the target server, and *resolver-level measurements*, where the application delegates DNS resolution to the Android system resolver. To observe the default behavior of the DNS queries issued by an application, we use the system resolver. However, when DNS resolution is delegated entirely to the system resolver, the protocol used depends on the Private DNS configuration of the device and on the resolver hostname, and therefore cannot be selected directly by the application. For this reason, comparing different DNS protocol versions requires controlling the query implementation at the application level.

**App-level Measurements:** In this configuration, we measure the behavior of the different DNS protocol versions under direct application control and, additionally, obtain the TLS session keys required to decrypt the captured encrypted traffic. To this end, the application implements five DNS protocol variants: Do53, DoT, DoH, DoH/3, and DoQ. Each variant requires its own dedicated library, as, to the best of our knowledge, no single Android library supports all five DNS protocol variants simultaneously. Do53 sends raw DNS wire-format packets [1] via DatagramSocket. DoT opens a TLS socket through the Conscrypt [58] TLS provider. DoH uses OkHttp’s DnsOverHttps client over an HTTP/2 connection. DoH/3 uses Google’s Cronet library [59] and DoQ uses the kwik QUIC library [60]. Although Google, Quad9, and AdGuard expose DoQ endpoints, DoQ was exercised only against Quad9 and AdGuard: at the time of the measurements, the Google DoQ server was not operational.

A property of the app-level configuration is that it provides direct access to the cryptographic material required to decrypt the traffic captures. Among the five protocol variants, only Do53 transmits queries in plaintext; DoT, DoH, DoH/3, and DoQ encrypt all DNS traffic. To obtain visibility into the encrypted payloads, the application exports TLS session keys in NSS SSLKEYLOGFILE format.

Two strategies are needed to obtain those keys, because the platform does not offer a single mechanism for all transports. For DoT and DoH we rely on Conscrypt rather than on the system BoringSSL library, which allows us to register a key-logging callback that stores the session keys as each TLS handshake completes; the BoringSSL version shipped with Android does not expose the symbols required to do this. For DoH/3 we use Cronet, which statically links its own BoringSSL instance and therefore cannot be instrumented in the same way, but which provides an internal key-logging facility, disabled by default, that writes the TLS secrets to a configured file path.

In this configuration, no DNS cache is used. Therefore, all queries within a benchmark target the same domain across all DNS protocol variants.

**Resolver-level Measurements:** In this configuration, we evaluate the default behavior of the DNS queries issued by an application when resolution is delegated to the Android system resolver, and we analyze how that behavior varies across different configured DNS servers. The application delegates DNS resolution entirely to the Android system resolver by invoking InetAddress.getAllByName(), which internally relies on the netd daemon. The behavior of the resolver is controlled through the Private DNS setting (Settings → Network & Internet → Private DNS). When a specific hostname is configured, the protocol used depends on the hostname: with dns.google (Google) and cloudflare-dns.com (Cloudflare) the resolver uses DoH/3, whereas with dns.quad9.net (Quad9) and dns.adguard-dns.com (AdGuard) it falls back to DoT, even though both servers support DoQ. This asymmetric behavior is implemented in the Android DnsResolver source code, which contains a hardcoded allowlist of hostnames that may be upgraded from DoT to DoH/3 [61]; for any hostname outside that list, the resolver remains on DoT regardless of server-side support.

In this configuration the application can neither select the protocol explicitly nor extract TLS session keys, since the encrypted connections are managed by netd and the corresponding key material is not exposed to user-space applications by Android. The system resolver also maintains an in-process DNS cache that is shared across applications and persists across benchmark runs. To prevent cache hits from masking the cost of an actual resolution, resolver-level benchmarks rotate the queried name through the Tranco top-domains list [62], a research-oriented ranking commonly used in DNS measurement studies, so that each query targets a different domain.

#### 3.3.3. Query Distribution Modes

The distribution of queries within the time window affects energy consumption, and is also relevant from the privacy point of view since dummy queries can be added either as a single burst or following a periodic pattern. If queries are sent in a single burst, the device radio and CPU are active for a short period and then return to idle. If queries are distributed periodically, the device may not fully enter low-power states between queries. To evaluate this effect, the application supports two benchmark modes, which we refer to as *burst mode* and *periodic mode* throughout the paper and in all figures. In burst mode, all queries are sent in rapid succession at the beginning of the time window, and the device remains idle for the remainder. In periodic mode, queries are distributed uniformly across the window with an inter-query interval of $T_{\mathrm{window}}/N_{\mathrm{queries}}$.

## 4. Experimental Evaluation

Having described the measurement system and the collected metrics, this section details the benchmarks performed. We divide the evaluation into two categories, app-level DNS resolution and resolver-level DNS resolution, because each level exposes a different set of characteristics that we are interested in measuring.

At the *app level*, since the application controls the protocol implementation, we can directly compare the behavior of the different DNS protocol variants (Do53, DoT, DoH, DoH/3, DoQ) on the same device, and also compare how each protocol behaves across different DNS servers. In addition, this configuration grants access to the TLS session keys, which allows us to decrypt the captured traffic and analyze its composition. At the *resolver level*, the resolution is delegated to the Android system resolver, which lets us observe the default DNS behavior of an application installed on the device. We also evaluate how this default behavior changes when the Private DNS setting is configured with the different DNS servers under study and, finally, we measure the effect of the system-wide DNS cache, which is only present in this mode.

### 4.1. Open-World Measurement Conditions

We measure in the open world, on a commercial smartphone connected to a live mobile network, so that the reported cost is the one a real deployment of the countermeasure would actually pay. Background application activity, operating-system power management, and radio state transitions are present in the readings. We treat these as the operating conditions of the defense rather than as noise to be eliminated, since a privacy mechanism deployed on a phone runs alongside these processes. We control instead the variability they introduce. To compare the measurements fairly, every run is performed at the same physical location, over the same mobile access network and with the device stationary, so that signal strength and radio state transitions are as homogeneous as possible across runs. No other application is used in the foreground during the experiments, the device is never connected to a charger during a run, and every configuration is repeated over ten independent runs so that sporadic background activity affects the dispersion of a measurement rather than its central value. All comparisons are made within the same device and under the same conditions, and we claim relative differences between protocols, resolvers, and scheduling strategies rather than absolute per-query energy figures, which are below the resolution of the BatteryStats subsystem.

### 4.2. App-Level DNS Resolution

In app-level mode, we observe the power consumption scaling with query rate, the differences in resolution time and a traffic analysis using the TLS session keys.

#### 4.2.1. Power Scaling with Query Rate

We measured the average power consumed by the mobile device for different query rates during a time window. The measurements were evaluated using five different variants of the DNS protocol and across 4 DNS servers. In this case, the time window is 2 min as an example of a time interval in which dummy queries would be added. The query rates are 100, 300 and 500 queries per time window; these rates were chosen because the aim is to detect any noticeable increase in power consumption rather than to obtain fine-grained per-request measurements. All measurements are performed using two distributions of the DNS queries within the time window: burst mode (drawn with a dashed line) and periodic mode (drawn with a dotted line) as shown in Figure 3.

Two main results stand out from Figure 3. The first one is the clear difference in average power consumption between burst and periodic modes: for the same total number of queries within a given observation window, the average power is consistently lower when the queries are sent as a single burst at the beginning of the window than when they are spaced periodically across it. This is because, after a burst, the device radio and CPU can return to low-power idle states for the remainder of the window, whereas under a periodic pattern the device is repeatedly woken up and prevented from staying in a deep idle state. The second observation is consistent with the protocol design considerations discussed in Section 2: Do53 and the more recent QUIC-based protocols (DoH/3 and DoQ) are the most efficient. Do53 consumes the least power, followed by DoH/3 and DoQ, while DoT and DoH are the protocols with the highest power consumption across all four servers. Table 4 reports the corresponding numerical values, so that the differences between protocols, servers, and distribution modes can be read quantitatively rather than by visual inspection of the figure.

**Statistical treatment.** Each configuration (distribution mode, server, protocol, and query rate) was measured in 10 independent runs and each point in Figure 3 is the mean of these runs. We then check whether this number of runs is sufficient to support the increase in power with the query rate shown in Figure 3. For this purpose, we compute the statistical power for each server, protocol, and distribution mode. The statistical power is the probability that a two-sided two-sample *t*-test (α=0.05) detects the observed difference between 100 and 500 queries per window, given the available runs and their dispersion. A power of at least 0.8, the conventional threshold [63], indicates that the number of runs is sufficient; a lower value indicates that it is not. Table 5 reports the result.

The power is at least 0.8 in 31 of the 36 cases, and the difference between 100 and 500 queries per window is significant in 35 of them. Four of the five cases below the threshold correspond to Quad9 and AdGuard in burst mode, which show a higher dispersion across runs than Google and Cloudflare. The remaining case, Do53 on Google in periodic mode, corresponds to the smallest increase in power between the two query rates. We do not interpret this higher dispersion as a lower reliability of these resolvers. It is consistent with the higher and more dispersed resolution times measured for Quad9 and AdGuard (Section 4.2.2 and Section 4.3.2): the longer and more variable a resolution is, the longer and more variable the fraction of each run during which the radio of the device remains in its high-power state. Part of this variability may come from the network path to each resolver; for instance, from our location, the minimum round-trip time to AdGuard (about 45–50 ms) is two to three times that of the other three resolvers (15–25 ms). However, our measurements do not allow us to isolate the cause.

#### 4.2.2. Per-Protocol Resolution Time Comparison

In addition to the power measurements, we analyzed the per-query resolution time of each protocol on each server. Figure 4 reports the resolution time of every protocol–server combination measured under burst mode with 100 queries per window. The resolution time correlates with the power consumption observed in Figure 3, Do53 is the fastest protocol, while DoT and DoH are the slowest, with DoH/3 and DoQ sitting in between.

#### 4.2.3. Per-Capture Traffic Analysis

Using the SSLKEYLOGFILE extracted during the runs, we decrypt the captured traffic and produce per-capture histograms of packets per minute. We focus this analysis on DoT, DoH, and DoH/3, since these are among the most widely deployed encrypted DNS transports. The goal is to identify which packet categories are exchanged by each protocol during a benchmark run. To this end, we decrypt the traffic and group the observed packets into protocol-specific categories along the trace.

For this comparison, we show three representative captures in which 50 queries were sent in periodic mode during a 1 min window. The resulting traces are summarized as stacked histograms of sent and received packets in 1 s bins, shown separately in Figure 5, Figure 6 and Figure 7.

In the DoT capture shown in Figure 5, beyond the initial handshake packets, the traffic is mainly composed of DNS requests and responses, ACK packets, and a small number of control packets. The DoH capture in Figure 6, in addition to the categories already seen in (DoT), also includes ping traffic throughout the trace. Finally, the DoH/3 capture in Figure 7 also contains QUIC stream packets.

Overall, DoH/3 exchanges more packet types, and often more packets in total, than DoT and DoH. This shows that packet count alone does not explain the energy results: despite its richer packet structure, DoH/3 remains more energy efficient than the TCP/TLS-based alternatives.

### 4.3. System-Level DNS Resolution

In system-level benchmarks, the application delegates DNS resolution to the system resolver. The application cannot select the protocol or extract TLS keys in this mode. Therefore, we evaluate the behavior of the Android system resolver when configured with different DNS servers via the Private DNS setting. We measure the power consumption scaling with query rate, observing the results both with and without cache, and the differences in resolution time.

#### 4.3.1. Power Scaling with Query Rate

Analogously to the app-level benchmarks, we also evaluated the average power consumed by the mobile device for different query rates during a fixed time window. However, the measurements were performed using the default behavior of each DNS server when configured through the Private DNS setting; therefore, only one protocol is observed per DNS server. We also use a 2 min observation window and the same query rates (100, 300, and 500 queries per window). All measurements are again performed under both burst and periodic distribution modes. The new parameter evaluated in system-level measurements is the cache effect: the system resolver uses an in-process DNS cache by default, so the measured power and latency depend on whether queries hit the cache or not.

**Without cache.** To observe the no-cache behavior, every query targets a different domain. We use the Tranco top-domains list [62] and rotate through it so that no name is repeated within a benchmark window. Figure 8 shows two subplots grouped by protocol: the left subplot reports the servers that the Android system resolver upgrades to DoH/3 (Google and Cloudflare), and the right subplot reports the servers that fall back to DoT (Quad9 and AdGuard). Burst mode is drawn with a dashed line style and periodic mode with a dotted line style. As observed in the figure, the behavior is similar to the app-level queries, since we use different domains to avoid cache usage.

**With cache.** To observe the cache effect, we restrict the queried set to only 100 unique domains, taken from the top of the Tranco list, and reuse them in a loop. At the time of these measurements, the Android 15 system DNS cache holds 64 entries, so a non-negligible fraction of the 100-domain set fits in the cache. Therefore, we expect a reduction in power consumption at the 300- and 500-queries-per-window rates, where the same domains are queried multiple times within the window. Figure 9 shows the analogous plots to Figure 8, this time with the cache active.

As observed in Figure 9, enabling the cache reverses the trend seen in the no-cache case: average power now decreases as the query rate increases, since most domains are already cached and no network activity is needed. For the DoH/3 servers (Google and Cloudflare), the maximum average power reaches approximately 1200 mW in periodic mode and around 600 mW in burst mode, which is roughly half of the power. The DoT servers (Quad9 and AdGuard) follow the same pattern but at slightly higher absolute values, peaking near 1400 mW in periodic mode and 700 mW in burst mode. In both cases, the cache yields a substantial energy saving, and the burst/periodic gap persists: sending queries in a single burst remains the more energy-efficient distribution strategy regardless of whether the cache is active. As in the app-level benchmarks, each system-level power value is also computed as the average of 10 independent samples, and the burst and periodic modes show analogous behavior in terms of measurement stability.

#### 4.3.2. Resolution Time Comparison

We also analyzed the per-query resolution time obtained at the system level. Since in this configuration there is a single protocol per server the comparison is reported per server, and we again show measurements both with and without cache. Figure 10 groups the results by the protocol selected by the system resolver: the left subplot shows Google and Cloudflare (both upgraded to DoH/3), and the right subplot shows Quad9 and AdGuard (both falling back to DoT).

The main results highlighted in Figure 10 are that enabling the cache reduces the resolution time for all servers, and that Quad9 and AdGuard show higher mean resolution times than Google and Cloudflare. Google and Cloudflare exhibit very similar behavior, while among the two DoT servers, AdGuard consistently shows higher resolution times than Quad9.

### 4.4. Discussion

The experiments above compared the five main DNS protocol variants (Do53, DoT, DoH, DoH/3, and DoQ) on four major public resolvers (Google, Cloudflare, Quad9, and AdGuard), at both the application level and the Android system-resolver level. This double perspective allows us to characterize, on the one hand, the intrinsic cost of each protocol when the application controls the implementation and, on the other hand, the actual behavior observed when DNS resolution is delegated to the system resolver under the Private DNS setting. In overall terms, beyond unencrypted Do53, DoH/3 is the most energy-efficient encrypted variant, and from our measurement vantage point in Madrid, Spain, the most efficient resolvers are Google and Cloudflare, which are also the ones that the Android system resolver upgrades to DoH/3.

Beyond protocols and resolvers, we also studied two query distribution strategies and the effect of the system-wide DNS cache. For the addition of dummy queries used by DQF, sending the dummy queries as a single burst is more energy-efficient than spreading them periodically across the observation window, since after a burst the device radio and CPU can return to deep idle for the rest of the window. The cache, in turn, plays a complementary role. It reduces the energy footprint by avoiding network activity for repeated names, and it also has a privacy consequence, although one that cuts both ways: fewer queries reach the wire, so less information is exposed to the resolver, but for the same reason a dummy query whose name is already cached never leaves the device and therefore provides no obfuscation at all. A countermeasure that draws dummy names from a small recurring set would be silently absorbed by the cache and would pay no energy while also buying no privacy.

**Effect of the measurement location.** All measurements were taken from Madrid, Spain, and the distance between the device and the nearest point of presence (PoP) of each resolver may vary the results in other geographical areas. Public resolvers are reached through anycast, so the node that answers a query, and hence the round-trip time and its variability, depends on the location of the client and on the routing of its mobile operator. Since the energy of a DNS resolution on a phone is dominated by the time the radio stays in a high-power state, a resolver whose nearest PoP is farther away, or reached through a more variable path, may induce a higher and more dispersed power consumption; from our location, this is the case of AdGuard, whose minimum round-trip time is two to three times that of the other resolvers.

### 4.5. Limitations

The results of this study should be interpreted with consideration of the following limitations. First, all experiments were performed with a single device model (Google Pixel 8 Pro), a single operating system version (Android 15), a single mobile operator, and a single location (Madrid, Spain). Other chipsets, modems, operating system versions, access technologies, and network conditions may change the absolute power values, so we do not claim that these values represent a typical user; our claims concern the relative differences between protocols, resolvers, and scheduling strategies measured under identical conditions. Second, BatteryStats is a coarse, 1 Hz estimator of the power drawn by the whole device rather than a precision instrument, which is why we report average power over an observation window instead of per-query energy. Third, resolution times were measured with a wall-clock timer of millisecond granularity, which limits the precision of the values obtained for queries answered from the system DNS cache (Section 3.3.1). Extending the measurements to multiple devices, network conditions, and vantage points, adopting a monotonic timing source, and using other measurement tools in addition to BatteryStats are part of our future work.

## 5. Conclusions

DNS is a fundamental Internet service that, although originally designed without privacy or security guarantees, has progressively been extended with privacy mechanisms such as DoT, DoH, DoH/3, and DoQ. Encrypting the channel, however, does not prevent the resolver at the other end of it from observing every queried name, nor does it prevent an on-path observer from fingerprinting activity from traffic metadata. This is what motivates client-side countermeasures such as the addition of dummy queries in our previous DQF proposal, in which the device emits additional dummy queries over its own DNS channel to distort the profile an adversary can build. Such countermeasures trade additional network activity for privacy, and on a smartphone that trade is paid in battery. This paper characterizes that cost across the full set of currently deployed DNS protocol variants on a real, unmodified Android device.

We addressed this gap by measuring, on a Google Pixel 8 Pro running Android 15, the average power consumption and the per-query resolution time of the five main DNS protocol variants (Do53, DoT, DoH, DoH/3, and DoQ) across four major public resolvers (Google, Cloudflare, Quad9, and AdGuard). We performed the measurements at two complementary levels, the application level (where our custom benchmark application controls the protocol implementation and exports TLS session keys) and the Android system-resolver level (where DNS is delegated to netd and configured through the Private DNS setting), using two query distributions: single burst and periodic. We additionally exploited the exported TLS keys to decrypt the captured traffic and inspect the categories of packets actually exchanged by DoT, DoH and DoH/3 protocols.

Our measurements show that, beyond unencrypted Do53, DoH/3 is the most energy-efficient encrypted DNS variant, and that, from our measurement location, Google and Cloudflare are the most efficient resolvers, which are also the ones that the Android system resolver upgrades to DoH/3 via Private DNS. DoT and DoH consistently consume the most power and exhibit the longest resolution times, while DoQ sits between DoH/3 and the TCP/TLS-based protocols. The two query distributions also have a clear and reproducible impact: sending dummy queries as a single burst is more energy-efficient than spreading them periodically across the window, because the device can return to deep idle for the rest of the window. Finally, the system-wide DNS cache provides both an energy saving and a reduction in the volume of network traffic.

Taken together, these results provide guidelines for the deployment of privacy-preserving DNS on mobile devices: DoH/3 should be preferred over DoT and DoH when energy is a concern, dummy queries should be added in bursts rather than periodically, since this reduces the energy paid for an identical privacy gain, and the system DNS cache should be leveraged for real queries while being accounted for when generating dummy ones, as a cached dummy name never reaches the wire and therefore provides no obfuscation. These conclusions were obtained with a single device, network, and location; while the protocol ranking follows from the design of each transport, the resolver ranking depends on the vantage point. As future work, we plan to extend the evaluation to additional devices, network conditions, and measurement locations, to adopt a monotonic timing source for the resolution-time measurements, to use other measurement tools in addition to BatteryStats, to integrate DQF logic for adding dummy queries into the system resolver in order to remove the application-level constraints, and to explore adaptive, battery-aware strategies for adding dummy queries that further reduce the energy overhead while preserving the privacy guarantees of the technique.

## Figures and Tables

**Figure 1:**
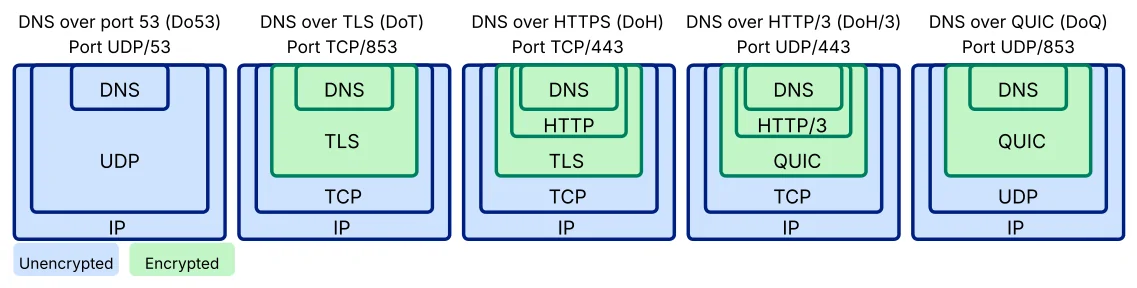
Packet-layer structure of the five DNS protocol variants considered in this work, starting from the IP layer.

**Figure 2:**
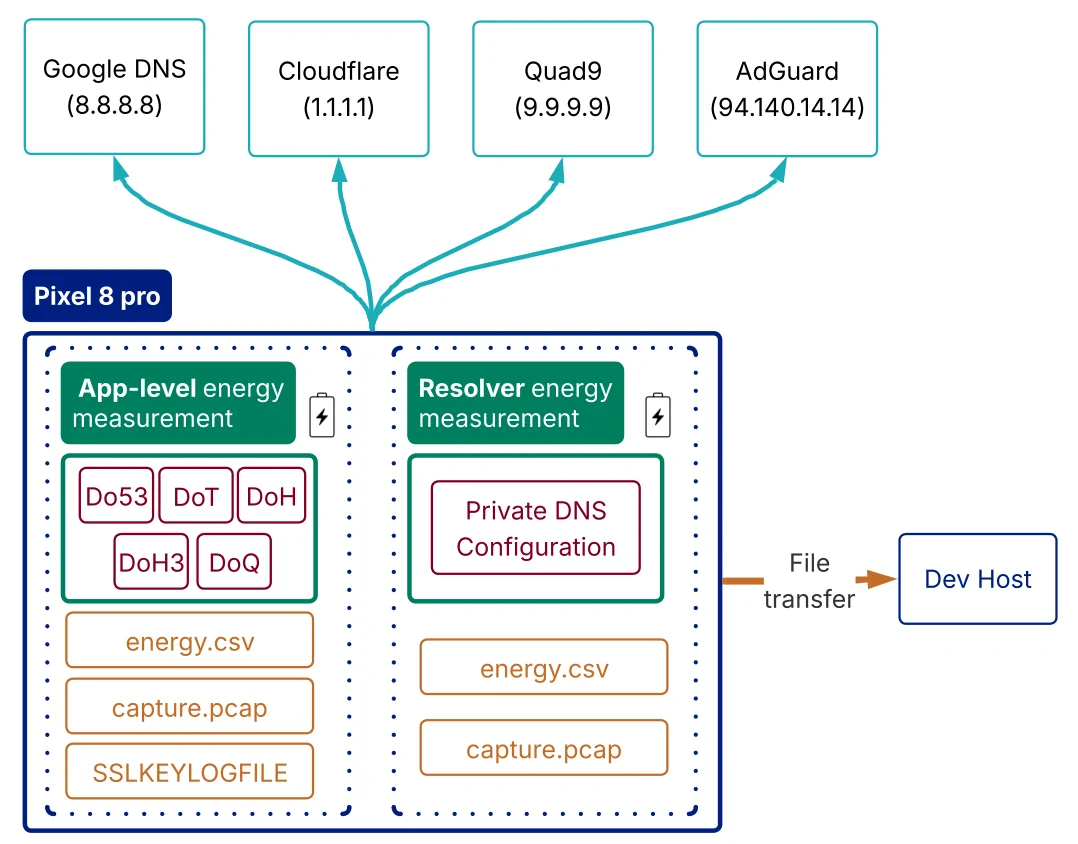
Testbed architecture for DNS energy measurement. Green: energy measurement; burgundy: DNS protocols; orange: output files; light blue: DNS servers; dark blue: devices.

**Figure 3:**
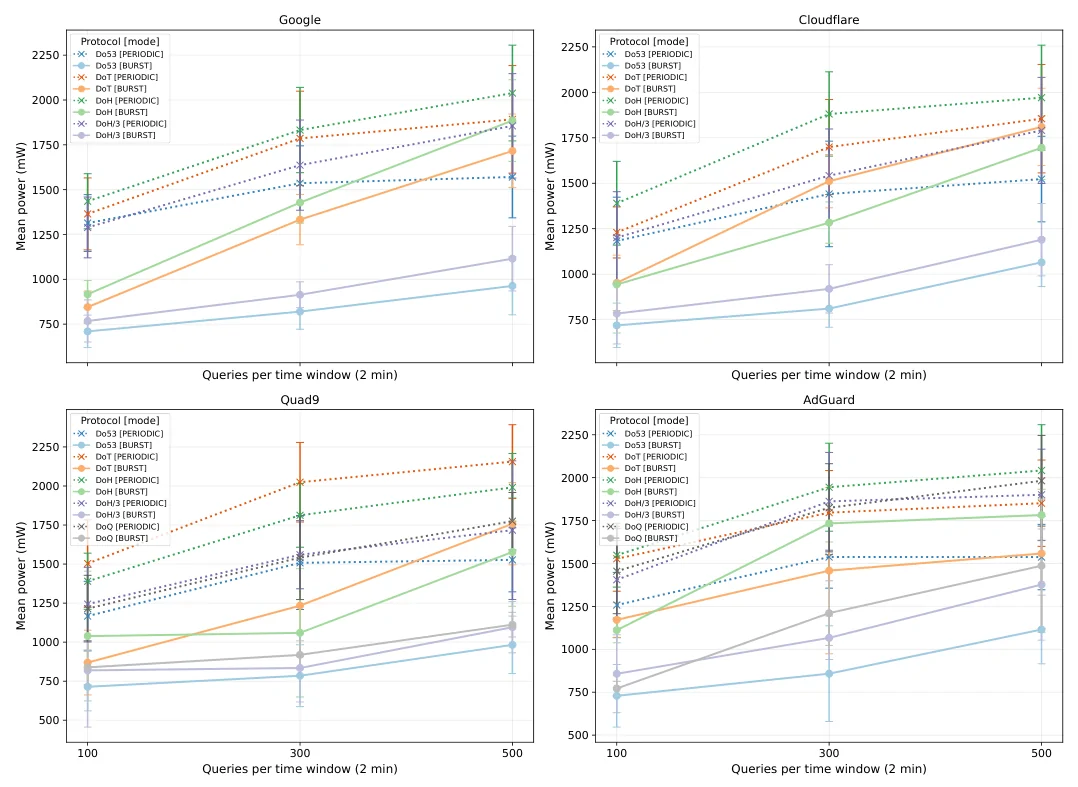
Average power consumption as a function of query rate for each DNS server with different versions of the DNS protocol: Google and Cloudflare with Do53, DoT, DoH, and DoH/3, and Quad9 and AdGuard with Do53, DoT, DoH, DoH/3, and DoQ; in burst mode (dashed lines) and periodic mode (dotted lines), measured over a 2 min observation window. For each rate, 10 iterations were executed.

**Figure 4:**
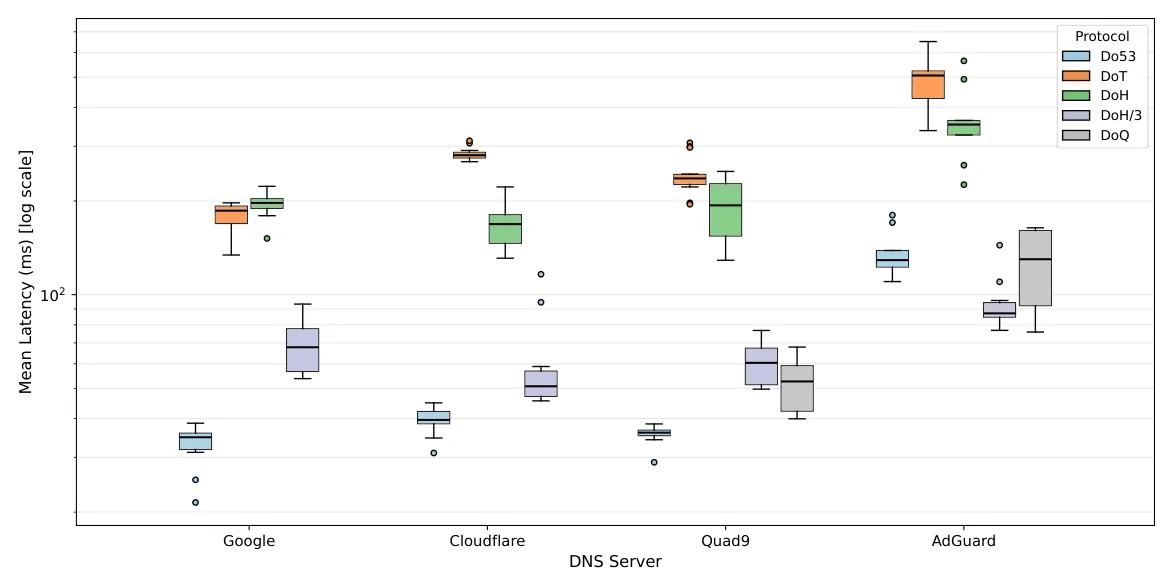
Per-query resolution time of each DNS protocol on each server (burst mode, 100 queries per run, log scale). Boxes span the interquartile range (IQR) with the median as the central line; whiskers extend to the most extreme samples within 1.5×IQR of the box, and dots represent the samples beyond this range.

**Figure 5:**
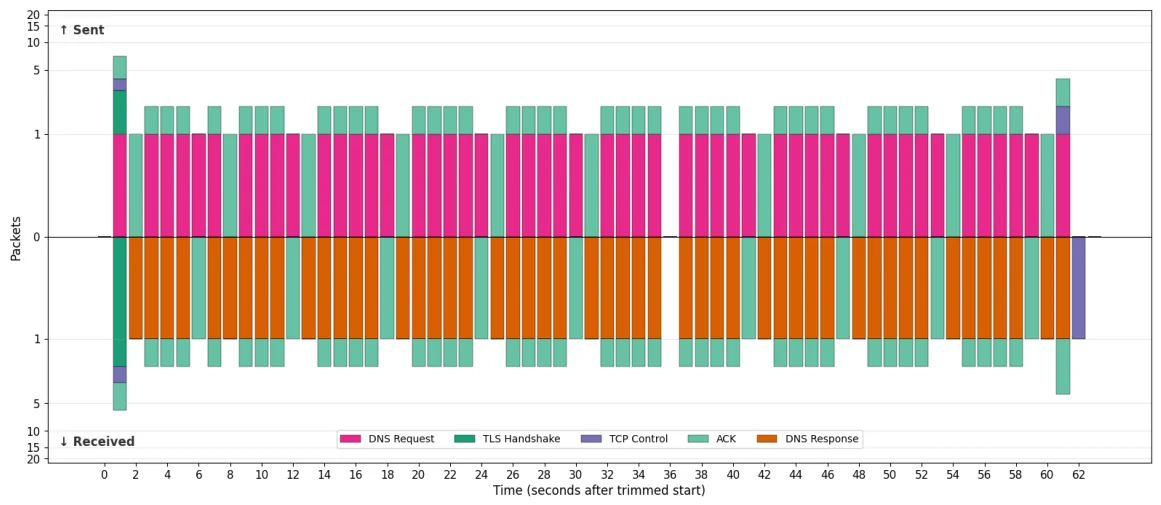
Per-capture stacked histogram of decrypted (DoT) traffic. The trace corresponds to 50 queries sent in periodic mode during a 1 min window, grouped into 1 s bins.

**Figure 6:**
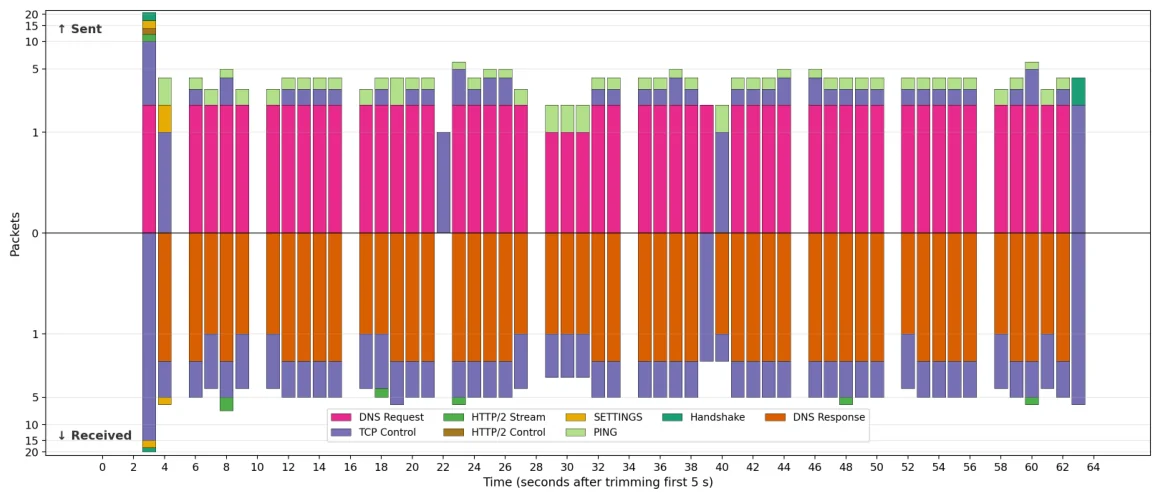
Per-capture stacked histogram of decrypted (DoH) traffic. The trace corresponds to 50 queries sent in periodic mode during a 1 min window, grouped into 1 s bins.

**Figure 7:**
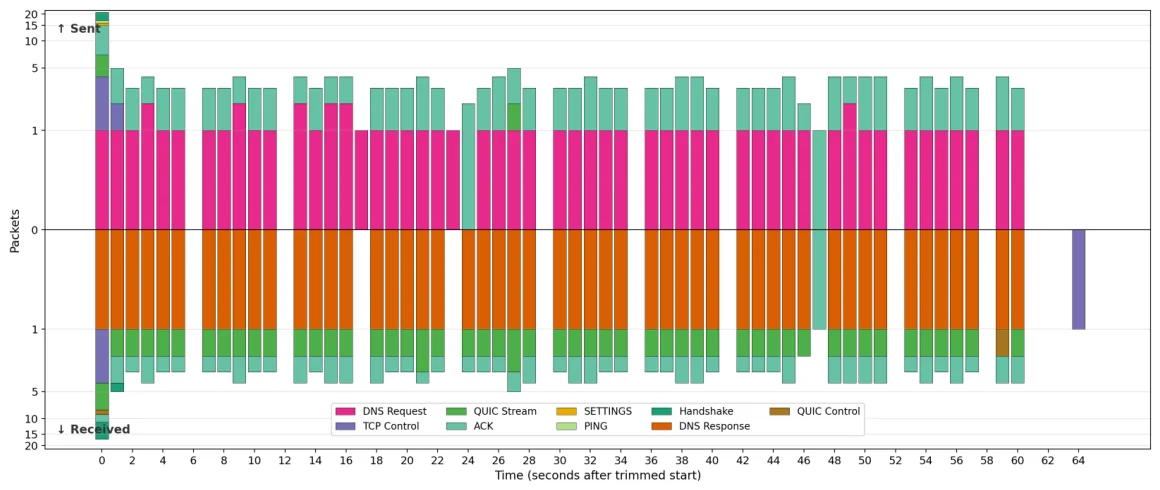
Per-capture stacked histogram of decrypted (DoH/3) traffic. The trace corresponds to 50 queries sent in periodic mode during a 1 min window, grouped into 1 s bins.

**Figure 8:**
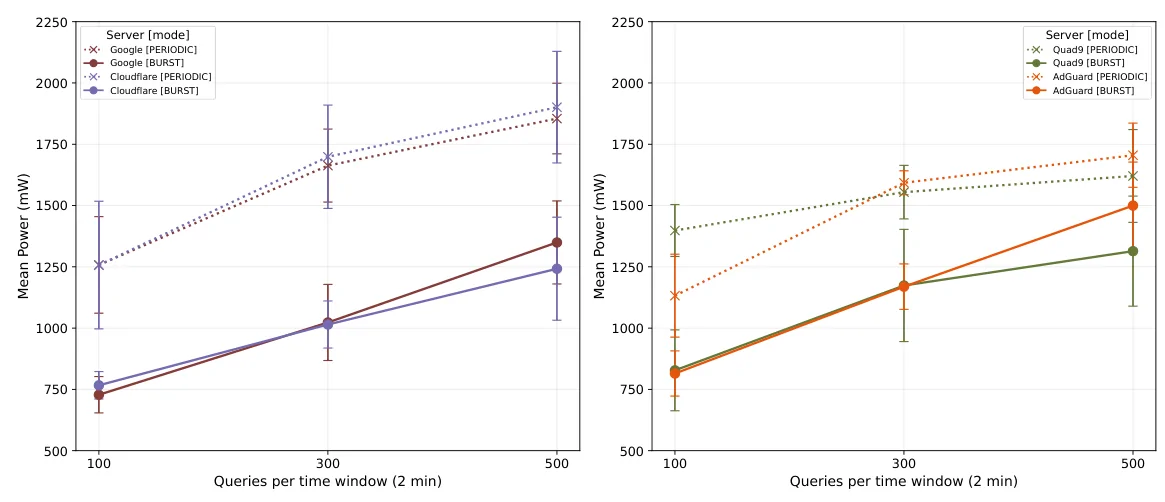
Average power consumption as a function of query rate at the system level, *without cache* (Tranco rotation). (**Left**): Servers using (DoH/3) (Google, Cloudflare). (**Right**): Servers using (DoT) (Quad9, AdGuard). Burst mode (dashed) vs. periodic mode (dotted), 2 min window.

**Figure 9:**
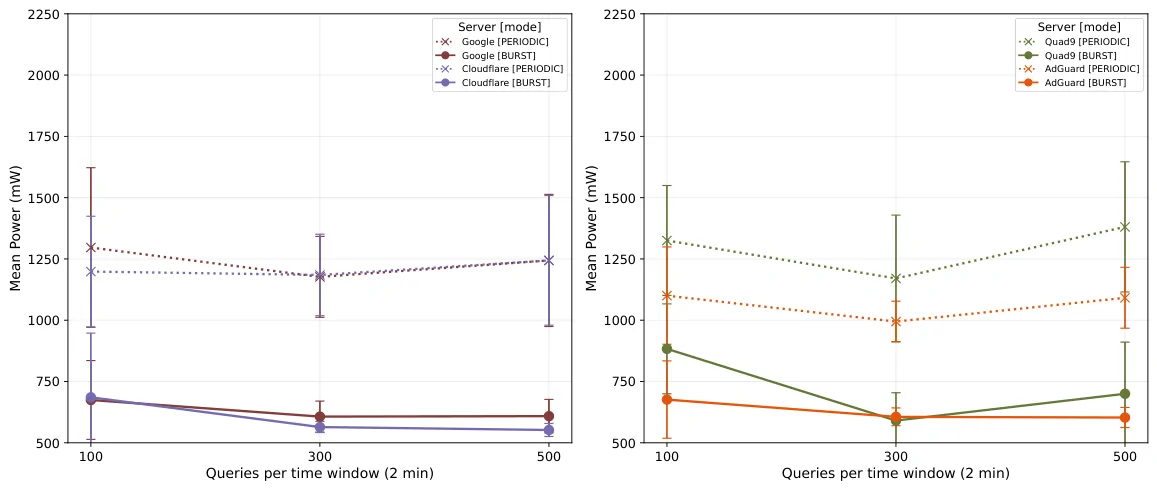
Average power consumption as a function of query rate at the system level, *with cache* (100 repeated domains from Tranco). (**Left**): Servers using (DoH/3) (Google, Cloudflare). (**Right**): Servers using (DoT) (Quad9, AdGuard). Burst mode (dashed) vs. periodic mode (dotted), 2 min window.

**Figure 10:**
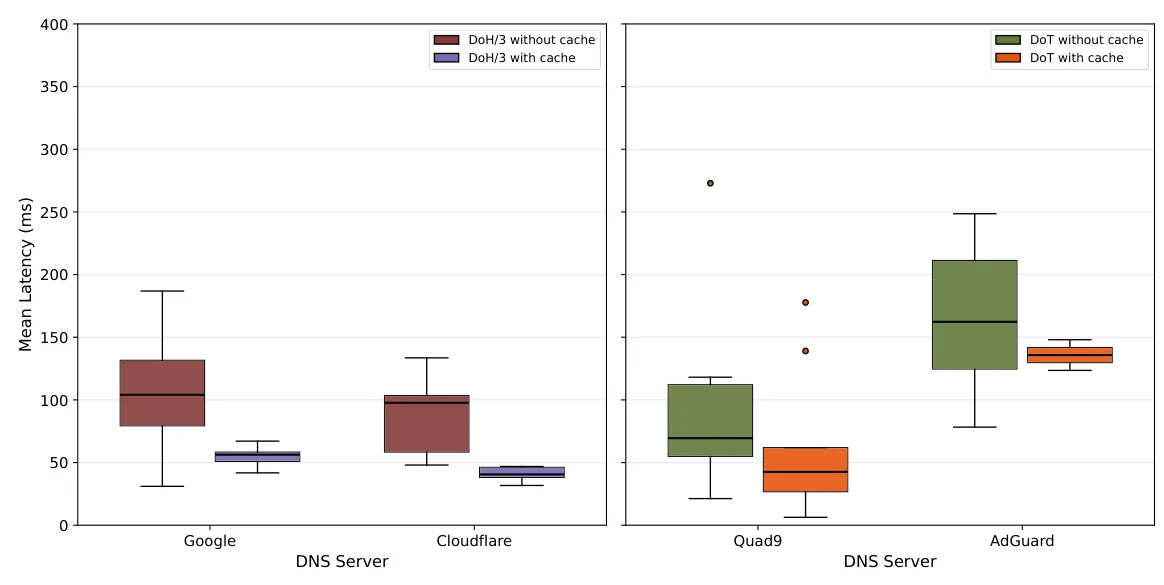
Per-query resolution time at the system level, with and without cache. (**Left**): Google and Cloudflare (DoH/3). (**Right**): Quad9 and AdGuard (DoT). Boxes span the interquartile range (IQR) with the median as the central line; whiskers extend to the most extreme samples within 1.5×IQR of the box, and dots represent the samples beyond this range.

**Table 1:** Evolution of DNS security and privacy protocols.

Ref.	Year	Protocol	Security Goal	Mechanism	Port
[4]	2005	DNSSEC	Integrity, authentication	Digital signatures on DNS records	any
[5]	2016	DoT	Confidentiality	TLS 1.3 on port 853	853
[33]	2016	QNAME Min.	Privacy (data minimization)	Minimal query name disclosure	any
[34]	2017	DNS-over-DTLS	Confidentiality (UDP)	DTLS for DNS transport	853
[6]	2018	DoH	Confidentiality, anti-censorship	DNS over HTTPS/HTTP2 on port 443	443
[8]	2022	DoQ	Confidentiality, low latency	DNS directly over QUIC	853
[7]	2022	DoH/3	Confidentiality, anti-censorship	DNS over HTTP3 on port 443	443
[9]	2022	ODoH	Confidentiality, anonymity	Proxy-based architecture	443

**Table 2:** Summary of studies measuring DNS protocol performance and energy costs.

Ref.	Year	Protocols	Parameters	Tool
[23]	2025	UDP, DoT, DoH	CPU power, RAM, energy	Yocto-Watt, Scaphandre
[41]	2023	UDP, TCP, DoT, DoH	CPU, memory, energy	Grid5000 cluster
[42]	2025	DNS, DNSSEC, DoT, DoH	Latency, traffic	BIND9, DNSPerf
[10]	2021	DoH, Do53	Resolution time	BrightData, 224 countries
[22]	2020	Do53, DoT, DoH	Query time, PLT	EC2, Firefox 67.0.1, getdns
[43]	2024	DoQ, DoH, DoUDP	PLT, handshake (RTTs)	Chromium 102.0.4991.0, CoreDNS 1.7.0
[44]	2025	DoQ, DoH/3, Do53	RTT, PLT	Firefox, RouteDNS
**This work**	2026	Do53, DoT, DoH, DoH/3, DoQ	Power, response time	Android 15, BatteryStats API, PCAPdroid v1.9.0

The row in bold corresponds to the present work.

**Table 3:** Summary of Android energy measurement tools and studies.

Reference	Year	Tool	Parameters
[45]	2010	Monsoon FTA22D	Power, State of Discharge
[46]	2025	Monsoon, iGreenMiner	Energy, Runtime, Power
[47]	2025	UM25C USB meter	Power, CPU utilization, Frequency
[48]	2024	ODPM, mobile devices	Power, Battery
[49,50]	2017	Batterystats, Systrace	Energy, Voltage, Intensity
[51]	2021	Batterystats, Battery Historian	Intensity, Power, Energy
[52]	2021	PETrA	Energy
[53]	2023	Android Runner/batterystats	Energy, CPU, Memory
[54]	2024	BatteryManager API, Lint	Battery capacity, Charge counter, Voltage, Intensity
[55]	2025	Snapdragon Profiler	CPU, Memory, Battery discharge
**This work**	2026	BatteryStats API, mobile device	Voltage, Intensity, Power, response time

The row in bold corresponds to the present work.

**Table 4:** Average power consumption (mW) at the application level for each server, protocol, distribution mode, and number of queries per 2 min window, corresponding to Figure 3: mean ± standard deviation over the runs, with the number of runs in parentheses.

		Burst			Periodic		
Server	Protocol	100	300	500	100	300	500
Google	Do53	710 ± 91 (10)	820 ± 99 (10)	963 ± 162 (10)	1314 ± 158 (9)	1536 ± 209 (9)	1571 ± 228 (9)
	DoT	845 ± 90 (10)	1333 ± 140 (10)	1716 ± 205 (10)	1365 ± 201 (10)	1786 ± 263 (10)	1892 ± 300 (10)
	DoH	917 ± 76 (10)	1428 ± 115 (10)	1885 ± 228 (10)	1435 ± 154 (10)	1832 ± 238 (10)	2039 ± 267 (10)
	DoH/3	768 ± 117 (10)	914 ± 72 (10)	1115 ± 180 (10)	1289 ± 170 (10)	1637 ± 252 (10)	1856 ± 291 (10)
Cloudflare	Do53	718 ± 122 (11)	811 ± 103 (11)	1065 ± 133 (11)	1183 ± 241 (11)	1441 ± 290 (11)	1523 ± 235 (11)
	DoT	952 ± 152 (12)	1511 ± 147 (12)	1810 ± 213 (12)	1229 ± 140 (12)	1699 ± 261 (12)	1855 ± 298 (12)
	DoH	943 ± 268 (12)	1283 ± 114 (12)	1695 ± 175 (12)	1390 ± 230 (10)	1881 ± 232 (10)	1971 ± 289 (10)
	DoH/3	783 ± 168 (12)	919 ± 133 (12)	1189 ± 199 (12)	1201 ± 252 (12)	1542 ± 256 (12)	1791 ± 292 (12)
Quad9	Do53	715 ± 155 (8)	785 ± 198 (8)	983 ± 184 (8)	1167 ± 223 (10)	1508 ± 299 (10)	1527 ± 205 (10)
	DoT	869 ± 207 (14)	1234 ± 327 (15)	1758 ± 263 (15)	1504 ± 280 (9)	2024 ± 255 (9)	2156 ± 236 (9)
	DoH	1039 ± 415 (8)	1060 ± 411 (9)	1578 ± 348 (9)	1390 ± 180 (10)	1812 ± 205 (10)	1991 ± 217 (10)
	DoH/3	820 ± 364 (9)	835 ± 218 (9)	1096 ± 164 (9)	1244 ± 235 (10)	1560 ± 218 (9)	1717 ± 444 (10)
	DoQ	839 ± 111 (6)	919 ± 90 (6)	1112 ± 79 (6)	1215 ± 213 (11)	1541 ± 269 (9)	1775 ± 184 (11)
AdGuard	Do53	729 ± 182 (10)	858 ± 279 (10)	1116 ± 200 (10)	1258 ± 190 (11)	1538 ± 182 (8)	1538 ± 191 (11)
	DoT	1172 ± 102 (11)	1459 ± 485 (11)	1558 ± 440 (11)	1528 ± 189 (11)	1797 ± 245 (11)	1851 ± 251 (10)
	DoH	1112 ± 74 (9)	1734 ± 108 (7)	1783 ± 150 (9)	1549 ± 186 (10)	1944 ± 256 (10)	2042 ± 267 (9)
	DoH/3	858 ± 227 (10)	1067 ± 127 (9)	1377 ± 324 (9)	1406 ± 229 (11)	1862 ± 285 (11)	1901 ± 265 (10)
	DoQ	772 ± 42 (6)	1211 ± 188 (6)	1487 ± 388 (6)	1455 ± 248 (15)	1825 ± 257 (15)	1982 ± 264 (15)

**Table 5:** Statistical power to detect the difference in average power between 100 and 500 queries per window, per server, protocol, and distribution mode (application level). Power: probability of detecting the observed difference with a two-sided two-sample *t*-test (α=0.05) and the available runs. ^†^ Power below the 0.8 threshold.

		Burst	Periodic
Server	Protocol	Power	Power
Google	DoH	1.00	1.00
	DoT	1.00	0.99
	DoH/3	1.00	1.00
	Do53	0.97	${0.70\;}^{†}$
Cloudflare	DoH	1.00	0.99
	DoT	1.00	1.00
	DoH/3	1.00	1.00
	Do53	1.00	0.87
Quad9	DoH	${0.70\;}^{†}$	1.00
	DoT	1.00	1.00
	DoH/3	${0.46\;}^{†}$	1.00
	Do53	${0.79\;}^{†}$	0.93
	DoQ	0.94	1.00
AdGuard	DoH	1.00	0.98
	DoT	${0.74\;}^{†}$	0.84
	DoH/3	0.91	0.98
	Do53	0.98	0.88
	DoQ	0.96	1.00

## Data Availability

The data and source code of the Android application is publicly available in https://github.com/perlab-uc3m/PowerConsumptionMeasurementsDNSqueries (accessed on 1 July 2026).

## Abbreviations

The following abbreviations are used in this manuscript:

DNS	Domain Name System
DNSSEC	DNS Security Extensions
Do53	Traditional DNS over UDP (port 53)
DoT	DNS over TLS
DoH	DNS over HTTPS
DoH/3	DNS over HTTP/3
DoQ	DNS over QUIC
ODoH	Oblivious DNS over HTTPS
DQF	DNS Query Forgery
PET	Privacy Enhancing Technique
KL	Kullback–Leibler
TLS	Transport Layer Security
QUIC	Quick UDP Internet Connections
IoT	Internet of Things
PQC	Post-Quantum Cryptography
API	Application Programming Interface
ADB	Android Debug Bridge
PLT	Page Load Time
RTT	Round-Trip Time
PoP	Point of Presence
SGX	Software Guard Extensions
TEE	Trusted Execution Environment
ODPM	On-Device Power Rails Monitor
